# Macular microvasculature in patients with thyroid-associated orbitopathy: a cross-sectional study

**DOI:** 10.1186/s13044-023-00175-3

**Published:** 2023-08-02

**Authors:** Mojtaba Abrishami, Aliakbar Sabermoghaddam, Zeinab Salahi, Elham Bakhtiari, Mehrdad Motamed Shariati

**Affiliations:** 1 Eye research center, Mashhad University of Medical Sciences, Mashhad, Iran; 2 Ocular Oncology Service, Department of Ophthalmology and Visual Sciences, University of Toronto, Toronto, Canada

**Keywords:** Thyroid-associated orbitopathy, Thyroid vasculopathy, Optical imaging, Macular flow density

## Abstract

**Purpose:**

The aim of this study was to evaluate macular blood flow in patients with thyroid-associated orbitopathy (TAO) as compared to healthy subjects. The inflammatory nature of the disease, as well as the vascular congestion caused by the increase in the volume of orbital soft tissue and extraocular muscles, rationalize the assessment of retinal blood flow changes in these patients.

**Methods:**

This is a cross-sectional study with the convenience sampling method. Macular flow density was assessed using optical coherence tomography angiography (OCTA) and compared between patients with TAO and healthy individuals. We also compared macular flow density in two subgroups of patients based on clinical activity score (CAS).

**Results:**

Eighty–five cases, including 30 healthy individuals and 55 patients with TAO, participated. The foveal avascular zone (FAZ) area was significantly larger in the patient group than in the control. Patients with active TAO with CAS 3 or more had significantly larger FAZ areas than those with CAS less than 3 (p = 0.04).

**Conclusion:**

We showed that the FAZ area is larger in active TAO patients and can be considered a possible candidate feature for monitoring disease activity and thyroid-associated vasculopathy.

## Introduction

The evaluation of macular microcirculation with the optical coherence tomography angiography (OCTA) method has recently become popular in systemic diseases affecting hemodynamics [1]. OCTA is a non-invasive, accessible, and relatively inexpensive method for monitoring macular blood flow. Previous studies have shown changes in macular blood flow in patients with hypertension, chronic kidney disease, hemodialysis, diabetes, and inflammatory diseases [2–4].

Thyroid-associated orbitopathy (TAO) is an inflammatory disorder involving intraorbital tissues. Due to inflammation and stimulation of adipogenesis, an increase in the volume of intraorbital fat and extraocular muscles occurs, which leads to compressive effects on the optic nerve as well as impaired venous drainage [5]. Mechanisms such as compartment syndrome and congestive optic neuropathy due to venous insufficiency can cause neuronal damage [6]. In addition, due to the inflammatory nature of the disease, local disturbances in microcirculation can lead to further impairment.

In this study, we aimed to evaluate changes in the macular blood flow in patients with TAO compared to healthy people.

## Methods

This is a cross-sectional study, with the convenience sampling method, and we aim to evaluate macular microcirculation in patients with TAO compared to healthy individuals. TAO was defined in the presence of at least two of the following: concurrent or recently treated immune-related thyroid dysfunction, typical ocular signs, and radiographic evidence of thyroid eye disease. We divided all participants into patients and control groups. The participants of the control group were called from the hospital staff who volunteered to participate in the study. All groups were matched for age and gender. Subjects with the following conditions were excluded from the study: previous ocular surgeries in the past 6 months, intraocular pressure > 20 mmHg, any history of glaucoma, age < 18 years old, refractive error more than 3 diopters of spherical equivalent, any diseases that can affect the neurovasculature of the retina such as uveitis or diabetes mellitus, pregnancy or breastfeeding, and consumption of oral contraceptive pills.

This study was carried out according to the Declaration of Helsinki, and the Mashhad University of Medical Sciences ethical committee approved this study (approval number: IRMUMSMEDICALREC.1399.260). We obtained informed consent from the participants.

All participants underwent best-corrected distance visual acuity (BCVA) measurement with thumbing E chart, slit-lamp biomicroscopy, Goldmann applanation tonometry, and complete dilated fundus examination (using a + 90D condensing lens). To determine the activity of TAO, we used the Clinical Activity Score (CAS) system [7], which includes the evaluation of 7 items as follows: spontaneous orbital pain, gaze-evoked orbital pain, eyelid swelling, eyelid erythema, conjunctival redness, chemosis, and inflammation of the caruncle or plica. Each item is rated one (if that item is positive) or zero. A total score of three or more is considered an active disease. According to this scoring system, we subdivided the patient group into two subgroups: the patients with CAS < 3 and the patients with CAS ≥ 3. All patients were biochemically euthyroid at the time of examinations.

**Fig. 1:**
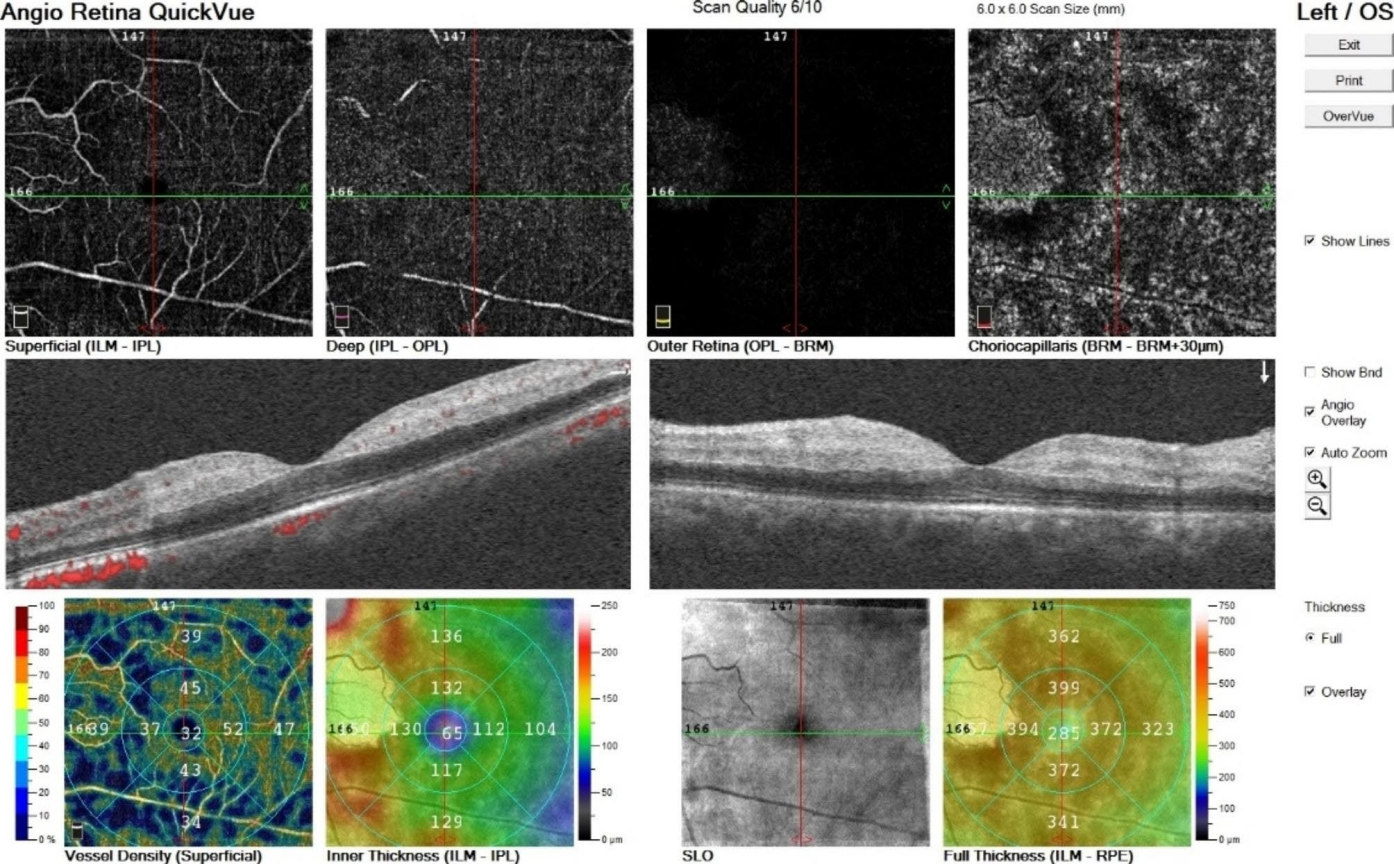
Representative image of the macular OCTA.

**Fig. 2:**
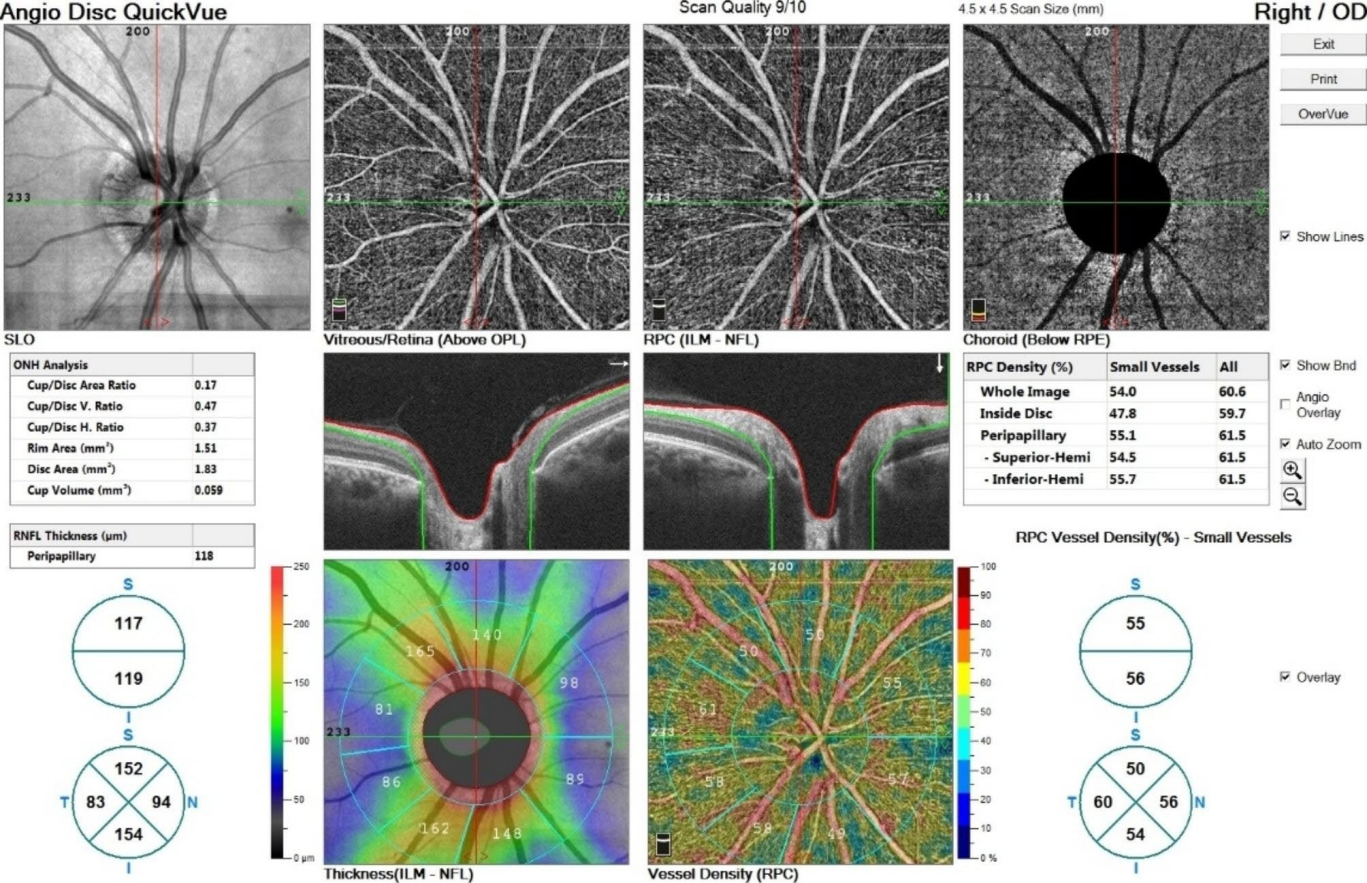
Representative image of optic nerve head OCTA.

All participants underwent macular optical coherence tomography angiography (AngioVue RTVue XR Avanti, Optovue, Fremont, CA, USA, software version: 2018,0,0,18) with 6 * 6 mm scan size. Superficial and deep capillary plexus were analyzed. Foveal vessel density (VD) was defined as the density of the superficial capillary plexus (internal limiting membrane to inner plexiform layer) in a 1 mm diameter circle centered on the center of the fovea. Parafoveal VD was defined as the ring occupying the area between the foveal area and the 2.5 × 2.5 mm area centered on the foveal center. Vessel densities were calculated automatically. Furthermore, the neuro-vasculature of the ONH was evaluated with OCTA imaging (AngioVue RTVue XR Avanti, Optovue, Fremont, CA, USA) of the disc. To detect the ONH vasculature an area of 4.5 × 4.5 mm centered on the (ONH) which included the papillary and peripapillary regions. To obtain the vascular density (VD) of the papillary area, the VD image was merged with the en-face OCT image of the ONH region with the built-in software of the device, and the papillary VD was calculated automatically. For the peripapillary area VD measurement, two circles were centered on the center of the ONH on the en face OCT image by a trained operator, one at the margin of the disc and the other at a radius of 2.25 mm from the center of the disc (4.5 mm diameter). The doughnut-shaped area made between them was defined as the peripapillary area. Radial peripapillary capillary (RPC) plexus density was measured both as a whole image (360 degrees around the ONH) and superior/inferior semicircles. Any images with a quality index below 6/10 were discarded, and the imaging was repeated (Figs. 1 and 2). Images were taken without any pharmacologic mydriasis and after 3–5 min of rest. All measurements were taken at 8–12 a.m. Any images with a quality index below 6/10 were discarded, and the imaging was repeated. The macular and peripapillary vascular profile includes foveal superficial and deep vessel density, parafoveal superficial and deep vessel density, foveal avascular zone (FAZ) area, radial peripapillary capillary (RPC) plexus density, and peripapillary retinal nerve fiber layer thickness (PRNFL) were analyzed and compared between the groups.

We used Statistical Package for Social Sciences (SPSS) software version 22 (IBM SPSS Statistics, IBM Corporation, Chicago, IL) for statistical analysis. We used the Shapiro-Wilk test to analyze the distribution of data. The characteristics of the subjects are described by descriptive statistical methods including central indices and indices of dispersion. We used the chi-square test to investigate the relationship between the qualitative variables and the independent samples t-test or its non-parametric equivalent to compare the quantitative variables between the groups. In all calculations, p < 0.05 was considered a significant level.

## Results

Eighty–five cases, including 30 healthy individuals and 55 patients with TAO, participated in the study. At the first examination, thirty-two patients had CAS < 3 and 23 patients had CAS ≥ 3. In this study, we analyzed the data from the right eye of each participant. Based on the Shapiro-Wilk test, the distribution of macular flow parameters was normal. Descriptive statistics of age, gender, and smoking were summarized in Table 1. All patients had Graves’ disease and were biochemically euthyroid with medications. None of our patients had any previous corticosteroid pulse therapy or radiation therapy for TAO. There was no statistically significant difference between the three groups in age, gender, and smoking. All of the subjects in both groups of patients and controls had a BCVA of 10/10, and there were no significant ocular conditions with potential impacts on retinal neurovasculature. The mean ± SD for intraocular pressure was 11.77 ± 1.41 mmHg and 11.18 ± 1.59 mmHg for the control and patient groups, respectively (P = 0.498).

### Foveal flow profile

While FAZ area was significantly larger in patients with TAO compared to healthy subjects (p = 0.020), foveal superficial and deep vessel density (SVD, DVD) profile evaluation indicated no difference between patients and the control group (Table 2a).

A comparison of flow data between the two subgroups of patients showedthat patients with active TAO with CAS 3 or more had significantly larger FAZ area than those with CAS less than 3 (p = 0.04). There was no statistically significant difference in other foveal vascular parameters between the two subgroups of patients (Table 2b).

### Parafoveal flow parameters

We found no significant difference between the two groups of patients and healthy subjects for the parafoveal flow parameters (Table 3a).

Although parafoveal deep vessel density was significantly lower in patients with CAS ≥ 3 compared to the other subgroup, the difference in the parafoveal superficial vessel density was not significant between the two subgroups (Table 3b).

**Table 1:** Demographics of all participants

Variable		Control group	Patients group		P-Value
			CAS < 3	CAS ≥ 3	
Age (years)(mean ± SD)		36.56 ± 2.33	44.34 ± 2.45	50.17 ± 3.21	0.23
Gender	Male (N, %)	12 (40)	7 (22)	6 (26)	0.71
	Female (N, %)	18 (60)	25 (78)	17 (74)	
Smokers (N, %)		2 (6)	7 (22)	3 (13)	0.40

CAS: Clinical Activity Score

**Table 2a:** Foveal flow profile in patients and controls

Foveal flow profile (percent)		Patients, Mean ± SD	Controls, Mean ± SD	P-value [95% Confidence Interval of the Difference]
	Foveal SVD	**15.95 ± 5.98**	**17.88 ± 6.76**	0.177 [-0.89, 4.86]
	Foveal DVD	**32.61 ± 6.27**	**33.24 ± 5.75**	0.653 [-2.06, 3.31]
	FAZ area	**0.36 ± 0.10**	**0.25 ± 0.07**	0.020^†^ [-0.05, 0.02]

^†considered statistically significant^

SVD: Superficial Vessel Density, DVD: Deep Vessel Density, FAZ: Foveal Avascular Zone

**Table 2b:** Comparison of foveal flow profile between the two subgroups of patients

Foveal flow profile (percent)		Patients with CAS ≥ 3, Mean ± SD	Patients with CAS < 3, Mean ± SD	P-value [95% Confidence Interval of the Difference]
	Foveal SVD	**16.92 ± 6.77**	**15.25 ± 5.35**	0.312 [-4.95, 1.61]
	Foveal DVD	**33.50 ± 7.24**	**31.98 ± 5.50**	0.379 [-4.96, 1.92]
	FAZ area	**0.33 ± 0.10**	**0.27 ± 0.11**	0.041^†^ [-0.02, 0.09]

^†considered statistically significant^

SVD: Superficial Vessel Density, DVD: Deep Vessel Density, FAZ: Foveal Avascular Zone

**Table 3a:** Parafoveal flow profile in patients and controls

Parafoveal flow profile (percent)		Patients, Mean ± SD	Controls, Mean ± SD	P-value [95% Confidence Interval of the Difference]
	Parafoveal SVD	**47.13 ± 7.5**	**49.91 ± 4.11**	0.064 [0.27, 5.28]
	Parafoveal SVD-temporal	**46.44 ± 7.44**	**48.49 ± 3.96**	0.165 [-0.42, 4.53]
	Parafoveal SVD-superior	**47.66 ± 7.85**	**51.02 ± 4.36**	0.034^†^ [0.71, 5.98]
	Parafoveal SVD-nasal	**46.08 ± 7.85**	**48.71 ± 4.17**	0.092 [0.03, 5.22]
	Parafoveal SVD-inferior	**48.41 ± 8.41**	**51.46 ± 4.64**	0.070 [0.23, 5.87]
	Parafoveal DVD	**53.76 ± 6.09**	**53.10 ± 3.92**	0.595 [-2.82, 1.51]
	Parafoveal DVD-temporal	**53.76 ± 6.85**	**53.47 ± 3.96**	0.830 [-2.64, 2.05]
	Parafoveal DVD-superior	**52.97 ± 7.23**	**52.52 ± 4.69**	0.759 [-3.30, 2.13]
	Parafoveal DVD-nasal	**54.37 ± 5.91**	**53.91 ± 3.41**	0.697 [-1.47, 2.55]
	Parafoveal DVD-inferior	**52.96 ± 6.72**	**52.53 ± 4.19**	0.754 [-2.78, 1.93]

^†considered statistically significant^

SVD: Superficial Vessel Density, DVD: Deep Vessel Density, FAZ: Foveal Avascular Zone

**Table 3b:** Comparison of parafoveal flow profile between the two subgroups of patients

Parafoveal flow profile (percent)		Patients with CAS ≥ 3, Mean ± SD	Patients with CAS < 3, Mean ± SD	P-value [95% Confidence Interval of the Difference]
	Parafoveal SVD	**46.30 ± 5.87**	**47.73 ± 8.52**	0.492 [-2.46, 5.32]
	Parafoveal SVD-temporal	**45.70 ± 5.09**	**46.99 ± 8.84**	0.534 [-2.54, 5.12]
	Parafoveal SVD-superior	**46.76 ± 7.16**	**48.32 ± 8.36**	0.472 [-2.65, 5.77]
	Parafoveal SVD-nasal	**45.74 ± 5.85**	**46.33 ± 9.11**	0.787 [-3.46, 4.64]
	Parafoveal SVD-inferior	**46.82 ± 7.83**	**49.55 ± 8.75**	0.239 [-1.78, 7.22]
	Parafoveal DVD	**51.70 ± 5.21**	**55.23 ± 6.33**	0.033^†^ [0.39, 6.66]
	Parafoveal DVD-temporal	**51.91 ± 6.79**	**55.13 ± 6.67**	0.088 [-0.49, 9.93]
	Parafoveal DVD-superior	**49.53 ± 6.92**	**55.45 ± 6.47**	0.002^†^ [2.26, 9.58]
	Parafoveal DVD-nasal	**52.70 ± 5.22**	**55.57 ± 6.17**	0.075 [-0.30, 6.05]
	Parafoveal DVD-inferior	**50.76 ± 6.25**	**54.54 ± 6.70**	0.039^†^ [0.24, 7.32]

^†considered statistically significant^

SVD: Superficial Vessel Density, DVD: Deep Vessel Density, FAZ: Foveal Avascular Zone

## Peripapillary flow parameters and RNFL thickness

We found a significant difference in whole image RPC density between the patients and control groups The PRNFL thickness in patients was lower than the controls. However, the difference was not statistically significant (Table 4a).

There was no significant difference in peripapillary flow parameters and RNFL thickness between the two subgroups of patients (Table 4b).

**Table 4a:** RPC density and PRNFL thickness in patients and controls

	Patients, Mean ± SD	Controls, Mean ± SD	P-Value [95% Confidence Interval of the Difference]
PRNFL thickness (µm)	**101.62 ± 21.17**	**103.83 ± 11.39**	**0.603 [-4.86, 9.28]**
Whole image RPC density (%)	**44.12 ± 7.23**	**47.09 ± 4.02**	**0.041** ^**†**^ **[0.13, 5.82]**
Whole image RPC density-superior hemisphere	**43.65 ± 7.31**	**46.99 ± 3.95**	**0.023** ^**†**^ **[0.90, 5.76]**
Whole image RPC density-inferior hemisphere	**44.58 ± 7.36**	**47.22 ± 4.13**	**0.075 [0.15, 5.11]**

^†considered statistically significant^

PRNFL: Peripapillary Retinal Nerve Fiber Layer, RPC: Radial Peripapillary Capillary

**Table 4b:** Comparison of RPC density and PRNFL thickness between the two subgroups of patients

	Patients with CAS ≥ 3, Mean ± SD	Patients with CAS < 3, Mean ± SD	P-Value [95% Confidence Interval of the Difference]
PRNFL thickness (µm)	**96.22 ± 19.37**	**105.50 ± 21.85**	**0.110 [-1.94, 20.50]**
Whole image RPC density (%)	**43.13 ± 5.76**	**44.83 ± 8.14**	**0.395 [-2.27, 5.67]**
Whole image RPC density-superior hemisphere	**42.78 ± 5.78**	**44.26 ± 8.27**	**0.459 [-2.53, 5.57]**
Whole image RPC density-inferior hemisphere	**43.44 ± 6.11**	**45.40 ± 8.13**	**0.333 [-1.89, 5.82]**

PRNFL: Peripapillary Retinal Nerve Fiber Layer, RPC: Radial Peripapillary Capillary

## Discussion

In this study, we evaluated retinal perfusion changes in patients with TAO compared to healthy individuals. The inflammatory nature of the disease, as well as the vascular congestion caused by the increase in the volume of orbital soft tissue and extraocular muscles, rationalize the assessment of retinal blood flow changes in these patients as a method to detect active subclinical cases [8–10]. In addition, systemic hemodynamic changes have been reported in dysthyroid rats [11]. Retinal blood flow is considered a potentially good model for the evaluation of systemic disturbances of circulation including hypertension, hypotension, heart failure, and following dialysis [12, 13].

Our results showed that the FAZ area was significantly larger in patients with TAO compared to healthy subjects (P < 0.05). Although evaluations of SVD and DVD at the fovea and parafovea showed a decrease in the patients’ group, this difference was not significant. The results of previous studies on macular perfusion in patients with TAO showed inconsistent results. While most of the studies has shown a decrease in foveal blood flow [14], others have shown contradictory results [10]. One study showed an increase in microvascular density at the macula in patients with active TAO as compared to healthy subjects [8]. Clinically, the higher FAZ area could potentially lead to lower visual function. However, we did not evaluate this hypothesis with functional visual assessment including contrast sensitivity, color vision, and perimetry.

Increased intraocular pressure, the presence of local inflammation, proptosis, smoking, and ischemia due to disturbances in venous drainage are the potential factors that may cause a decrease in retinal vascular density in patients with TAO [15, 16]. In this study, we did not investigate inflammatory biomarkers in patients, but a significant inverse correlation between serum levels of thyroid-stimulating hormone-receptor autoantibodies and macular microperfusion has been shown in a study [17]. One hypothesis that can be proposed as a possible pathogenic mechanism in retinal blood flow disturbances is thyroid-related vasculopathy, and immune-related vessel wall thickening [18].

Our results showed a significant increase in the FAZ area in patients with a more active stage of TAO (CAS ≥ 3) compared to those with inactive disease (CAS < 3). The correspondence of the FAZ area status with the patients clinical score makes it possible to use this parameter in estimating the clinical status of the patient as an auxiliary method. Besides, parafoveal deep vessel density was significantly lower in patients with CAS ≥ 3 compared topatients with CAS < 3. Considering no significant difference in superficial vessel density between these two subgroups was found, it can be concluded that with the increase in the activity of TAO, the deep flow density is more affected. The differences in vessel diameter and regional autoregulatory mechanisms between the superficial and deep capillary plexus of retina may be the cause of this discrepancy. However, most of the previous studies have shown a decrease in vascular density in both the superficial and deep layers of the retina with increasing severity of clinical involvement [19–21].

In this study, we found a statistically significant lower RPC density in TAO compared to healthy subjects, which is compatible with the results of some previous studies [22].

Del Noce et al. evaluated the changes in the macular flow density 2 months following intermittent intravenous infusion of high-dose corticosteroids (pulse therapy) in TAO patients. They showed that, along with the improvement of the clinical score, macular vascular flow density increased in patients [23]. The reversibility of flow density changes with treatment makes the assessment of macular vascular flow a potentially useful tool in monitoring TAO.

This study has several limitations. The sample size was small and the design of the study was cross-sectional. Besides, we did not evaluate the effects of treatment on the retinal flow profile in patients. Using functional tests such as perimetry and comparing the relation of macular vascular profile with visual field defects can be helpful in the evaluation of the precedence or latency of structural and functional changes. Due to the possibility of the influence of the hormonal differences on the vasomotor condition between the two sexes, the failure to compare flow indices between the two sexes, due to the small sample size, is one of the shortcomings of this study. Another limitation was the inability to analyze the effect of cigarette smoking as a confounding factor, although there was no significant difference between the study groups regarding this variable. In this study, we did not evaluate the changes in macular thickness profile including inner retinal thickness and ganglion cell complex thickness in TAO, which could be helpful in determining the synchronicity of vascular dropout and neuronal loss in clinically active patients [22].

## Conclusion

We showed that the FAZ area is larger in patients with active TAO than in healthy controls and it can be considered a possible feature for monitoring the activity of TAO and thyroid-associated vasculopathy.

## Data Availability

The data that support the findings of this study are available from the corresponding author upon reasonable request.
